# Eye Movements Discriminate Fatigue Due to Chronotypical Factors and Time Spent on Task – A Double Dissociation

**DOI:** 10.1371/journal.pone.0087146

**Published:** 2014-01-22

**Authors:** Dario Cazzoli, Chrystalina A. Antoniades, Christopher Kennard, Thomas Nyffeler, Claudio L. Bassetti, René M. Müri

**Affiliations:** 1 Nuffield Department of Clinical Neurosciences, University of Oxford, John Radcliffe Hospital, Oxford, United Kingdom; 2 Perception and Eye Movement Laboratory, Inselspital, Bern University Hospital, Departments of Neurology and Clinical Research, University of Bern, Bern, Switzerland; 3 Center of Neurology and Neurorehabilitation, Department of Internal Medicine, Luzerner Kantonsspital, Luzern, Switzerland; University of Ulm, Germany

## Abstract

Systematic differences in circadian rhythmicity are thought to be a substantial factor determining inter-individual differences in fatigue and cognitive performance. The synchronicity effect (when time of testing coincides with the respective circadian peak period) seems to play an important role. Eye movements have been shown to be a reliable indicator of fatigue due to sleep deprivation or time spent on cognitive tasks. However, eye movements have not been used so far to investigate the circadian synchronicity effect and the resulting differences in fatigue. The aim of the present study was to assess how different oculomotor parameters in a free visual exploration task are influenced by: a) fatigue due to chronotypical factors (being a ‘morning type’ or an ‘evening type’); b) fatigue due to the time spent on task. Eighteen healthy participants performed a free visual exploration task of naturalistic pictures while their eye movements were recorded. The task was performed twice, once at their optimal and once at their non-optimal time of the day. Moreover, participants rated their subjective fatigue. The non-optimal time of the day triggered a significant and stable increase in the mean visual fixation duration during the free visual exploration task for both chronotypes. The increase in the mean visual fixation duration correlated with the difference in subjectively perceived fatigue at optimal and non-optimal times of the day. Conversely, the mean saccadic speed significantly and progressively decreased throughout the duration of the task, but was not influenced by the optimal or non-optimal time of the day for both chronotypes. The results suggest that different oculomotor parameters are discriminative for fatigue due to different sources. A decrease in saccadic speed seems to reflect fatigue due to time spent on task, whereas an increase in mean fixation duration a lack of synchronicity between chronotype and time of the day.

## Introduction

Fatigue is generally defined as a sensation of weariness and drowsiness, inhibiting or impairing activity, and leading to a reduced desire of physical or mental effort [1], [2]. In healthy individuals, acute fatigue has a substantial negative impact on cognitive performance [3], [4]. For instance, fatigue is the most important identifiable cause of transport operations accidents [5]. In clinical populations, chronic fatigue is a symptom accompanying a variety of neurological conditions (for a review, see [6]).

In healthy subjects, systematic differences in circadian rhythmicity are thought to be one of the most substantial factors determining inter-individual differences in fatigue and performance [7]. In particular, the existence of physiological and behavioural differences between morning types (often called ‘larks’) and evening types (often called ‘owls’) at different times of the day has been recognised [8]. Morning types have a preference of getting up and going to bed early, with rather rigid sleep patterns. Evening types prefer to go to bed late in the night and generally have difficulties in getting up in the morning [9].

Optimal cognitive performance is reached when the time of testing coincides with the respective circadian peak period (i.e., morning hours for morning types and evening hours for evening types), a phenomenon referred to as synchronicity effect [10]. Although fluctuations in performance depending on the synchronicity effect are well known to affect a broad range of cognitive functions, they are rarely systematically assessed (for a review, see [11]).

Eye movements have been shown to be a reliable indicator of fatigue. Schmidt et al. [12] have been among the first to quantitatively consider the effects of mental and muscular fatigue upon saccade velocity. Decreased saccadic speed was then consistently found in healthy controls during continuous wakefulness or sleep deprivation [13], [14], [15], [16], [17]. Furthermore, eye movements can be measured continuously during extended cognitive tasks, allowing the assessment of the influence of fatigue during such tasks. Unlike other saccadic parameters, saccadic speed has been shown not to be subjected to voluntary control and therefore might be a more accurate representation of the underlying neural activity [18], [19]. A decrease in saccadic speed has also been found as a function of fatigue due to the increasing time spent on cognitive tasks [20], [21], [22]. Moreover, a decrease in saccadic speed has been recently shown to correlate with the subjectively experienced fatigue in patients suffering from multiple sclerosis [23]. On the other hand, increasing time spent on task did generally not affect the duration of visual fixations [22], [24], [25]. However, to the best of our knowledge, eye movements have not been used as a tool for the investigation of inter-individual differences in circadian rhythmicity and the resulting differences in fatigue.

The aim of the present study was to assess the effects of two factors that may potentially influence fatigue. First, we evaluated the effects of the lack of synchronicity between chronotype and time of the day (optimal or non-optimal); second, we assessed the influence of the time spent on task. The outcome was quantified by means of several eye movement parameters, considering both saccades and fixations.

## Materials and Methods

### Participants

Participants were recruited by poster announcements and word-of-mouth, asking for ‘morning people’ and ‘evening people’. All responders were assessed with the Morningness-Eveningness-Questionnaires (German version, D-MEQ, [26]; originally developed in English by Horne & Östberg [9]). Eighteen individuals were included in the study, i.e., the first nine responders whose chronotype corresponded to ‘definitely morning types’ and the first nine responders whose chronotype corresponded to ‘definitely evening types’. According to the cut-off scores set by Horne and Östberg (1976), ‘definitely morning types’ (henceforth referred to as morning types) had a D-MEQ score of ≥70, and ‘definitely evening types’ (henceforth referred to as evening types) had a D-MEQ score of ≤30. The nine morning types (four women) had a mean age of 27.56 years (standard deviation [SD] = 10.86) and the nine evening types (three women) had a mean age of 22.22 years (SD = 1.99). There was no significant age difference between the two groups (*t*_16_ = 1.449, *p* = .167, two-tailed). All participants were right-handed and had normal or corrected-to-normal visual acuity.

### Ethics Statement

All participants gave their written informed consent prior to the onset of the experiment and after the procedure had been explained to them. The study was consistent with the latest Declaration of Helsinki and was approved by the Ethical Committee of the State of Bern.

### Visual Stimuli

The visual stimuli consisted of 192 full-colour pictures depicting both rural and urban landscapes. The pictures were presented full-screen, subtending approximately 29×22° of visual angle at a viewing distance of 70 cm.

### Apparatus

A cathode ray tube display (Samsung SyncMaster 959NF) was used for the presentation of the stimuli, with 24-bit colour depth and a refresh rate of 85 Hz. The screen (36×27 cm) had a resolution of 800×600 pixels.

Eye movements were recorded in a dimly lit room, using an infrared, video-based eye-tracking system (Eyelink™, Sensomotoric Instruments GmbH, Teltow, Germany). The system has a temporal resolution of 250 Hz, a spatial resolution of 0.01°, and a typical gaze position accuracy of 0.5–1° (largely depending on the calibration precision).

The parsing algorithm of the system was set to detect saccades when the eye moved at least 0.1° and either eye speed exceeded 35°/s or acceleration exceeded 9500°/s^2^. Due to the spatial resolution of the system, saccades with an amplitude <1° were excluded from the analysis. The mean speed of every individual saccade was computed as the average of the instantaneous saccadic speeds in every sample of 4 msec (given a 250 Hz sampling rate). Saccadic speed has to be understood as vector velocity, where the instantaneous saccadic speed in every sample is calculated as the Euclidian sum of its x and y components.

The head of the subjects was stabilised using a chin-rest. The system was periodically calibrated by means of two 3×3 points grid calibration sequences.

### Procedure

All participants took part in two testing sessions at two different times of the day: a) in the morning between 8am and 9am (optimal time for the morning types and non-optimal time for the evening types); and b) in the evening between 5pm and 6pm (optimal time for the evening types and non-optimal time for the morning types) (similarly to [27]). The two testing sessions took place on the same weekday of two consecutive weeks, and the order of the sessions was counterbalanced over participants.

First, participants were asked to rate their current level of fatigue on a visual analogue scale (VAS), i.e., to draw a vertical stroke on a 100 mm horizontal line, ranging from ‘I’m not fatigued at all’ to ‘I’m extremely fatigued, exhausted’.

Second, participants performed the free visual exploration task while their eye movements were recorded. The 192 pictures were randomly allocated to six blocks, yielding 32 pictures in each block. The order of the pictures within each block was randomised differently for each participant. Each picture was presented for 10 s and was preceded by a central fixation point for 1.5 s. The central fixation point enforced a common starting point of visual exploration and allowed drift correction of the eye-tracking system data. Each free visual exploration block lasted approximately 6 minutes. The participants were instructed to initially fixate at the central fixation point and then to freely explore the pictures. Participants completed the free visual exploration task of three out of the six blocks of pictures for each testing session. The three blocks of pictures and their order were randomly chosen for the first and the second session, respectively (i.e., the participants saw each block of pictures just once during the whole experiment). Between blocks, a brief break of about one minute allowed for the recalibration of the eye-tracking system. Each testing session lasted in total about 25 minutes.

### Data Analysis

For the VAS, the distance of the vertical stroke from the left extreme of the horizontal line was measured in mm. The score could therefore range from 0 to 100, and the higher the score, the more pronounced the subjective fatigue. The results were analysed by means of a repeated-measures analysis of variance (ANOVA) with the between-subjects factor ‘chronotype’ (morning types, evening types) and the within-subjects factor ‘time of the day’ (optimal, non-optimal).

Individual saccades on each picture were characterised by their mean speed (see Apparatus section). The average of these measures was then calculated for each picture, and finally a grand average was calculated for each block. Analogously, the average duration of the individual visual fixations on each picture was computed, and finally a grand average was calculated for each block. The resulting variables were named ‘mean saccadic speed’ and ‘mean fixation duration’, respectively. The results were analysed by means of repeated-measures ANOVAs with within-subjects factors ‘time of the day’ (optimal, non-optimal) and ‘block’ (1, 2, 3). To control for the effects of the chronotype *per se*, these analyses were also rerun with the additional between-subjects factor ‘chronotype’ (morning types, evening types).

To follow-up on the relation between subjectively estimated fatigue and mean fixation duration at optimal or non-optimal times of the day, a Pearson’s correlation was calculated between the difference of the VAS scores (i.e., VAS score at non-optimal time - VAS score at optimal time) and the difference of the mean fixation durations (i.e., mean fixation duration at non-optimal time - mean fixation duration at optimal time; both values averaged over the three blocks).

To control for the confounding effect of saccadic amplitude on saccadic speed, the mean saccadic amplitude data were analysed by means of a repeated-measures ANOVA with within-subjects factors ‘time of the day’ (optimal, non-optimal) and ‘block’ (1, 2, 3).

*Post-hoc* comparisons were computed by means of Fisher’s least significant difference-corrected *t*-tests.

## Results

**Figure 1:**
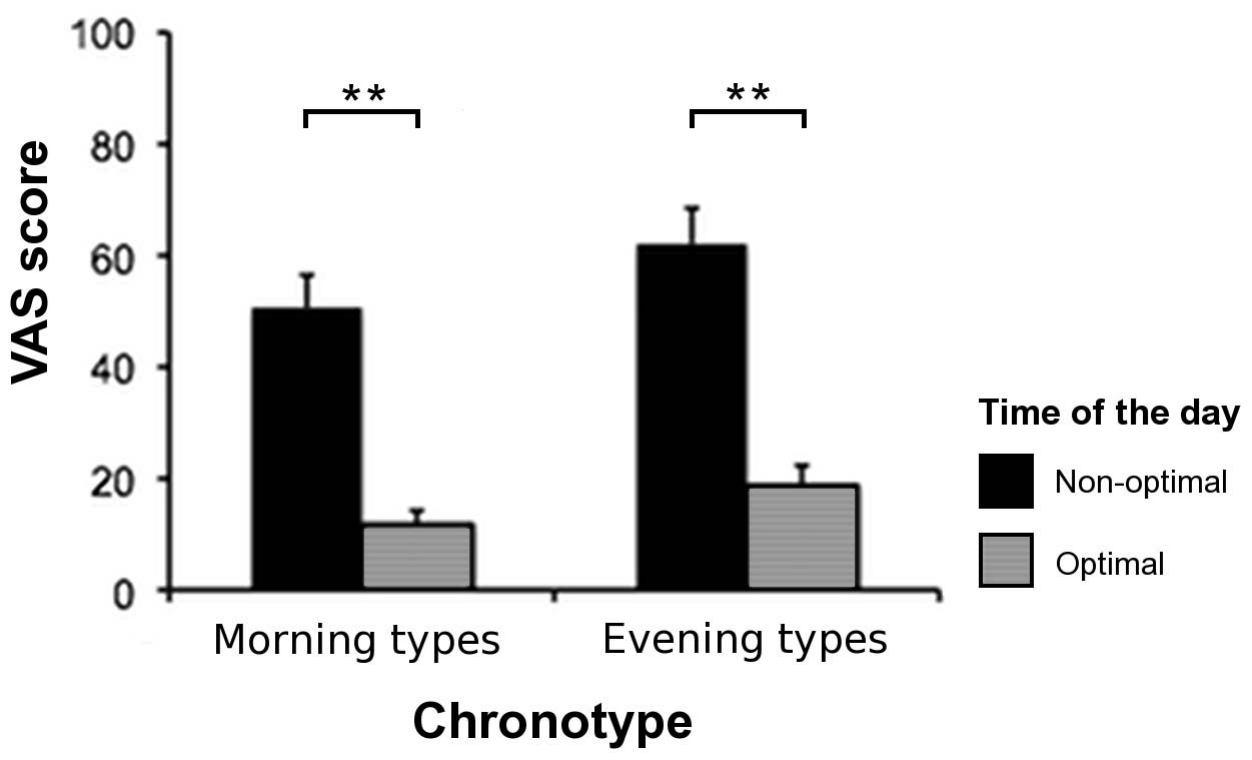
Mean visual analogue scale (VAS) fatigue scores. Mean VAS scores reflect subjective fatigue of the participants according to their chronotype (morning or evening types) and the time of the day (non-optimal or optimal). Error bars represent the standard error of the mean (SEM). Asterisks depict significant post-hoc tests (**p<.01).

Concerning the VAS, the repeated-measures ANOVA revealed a significant effect of the factor ‘time of the day’ (F_1,16_ = 33.59, *p*<.001), but not of the factor ‘chronotype’ (F_1,16_ = 1.93, *p* = .18) or of the interaction ‘chronotype × time of the day’ (F_1,16_ = .1, *p* = .76). That is, participants subjectively reported in the VAS a more pronounced fatigue at their non-optimal than at their optimal time of the day, irrespectively of their chronotype (see Fig. 1).

**Figure 2:**
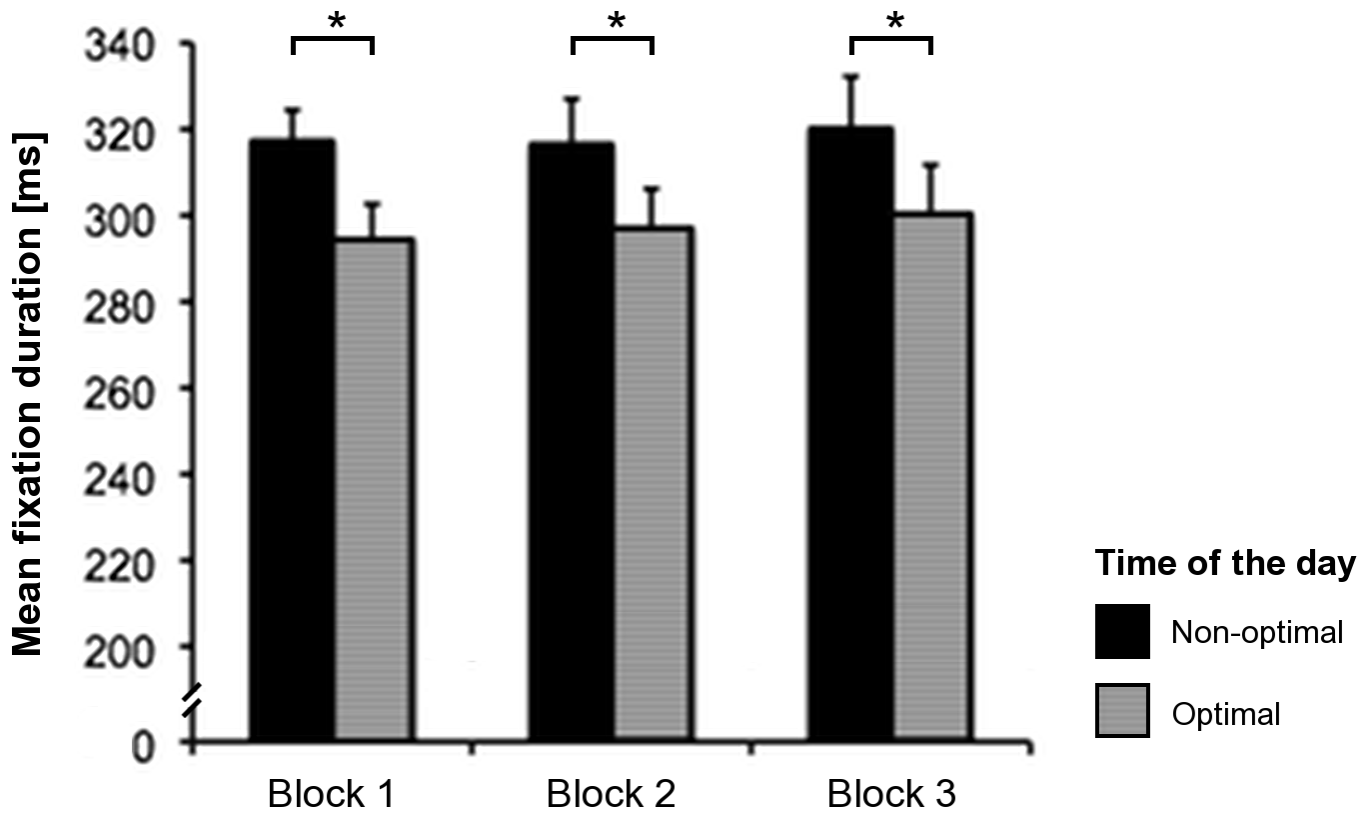
Mean fixation duration. Mean fixation duration in the three blocks of the free visual exploration task, as measured at optimal and non-optimal times of the day for the participants. Error bars represent the standard error of the mean (SEM). Asterisks depict significant post-hoc tests (*p<.05).

For the mean fixation duration during the free visual exploration, the repeated-measures ANOVA revealed a significant effect of the factor ‘time of the day’ (F_1,17_ = 8.64, *p* = .009), but not of the factor ‘block’ (F_2,34_ = .24, *p* = .79) or of the interaction ‘time of the day × block’ (F_2,34_ = .05, *p* = .95). The mean fixation duration during the free visual exploration was significantly longer at the non-optimal than at the optimal time of the day, and this difference remained the same during the whole task (see Fig. 2). Rerunning the repeated-measures ANOVA with the additional factor ‘chronotype’ yielded the same results, i.e., the only significant effect was found for the factor ‘time of the day’ (F_1,16_ = 8.18, *p* = .011), whereas the effects of the other factors or interactions were not significant (‘block’: F_2,32_ = .24, *p* = .79; ‘chronotype’: F_1,16_ = .48, *p* = .5; ‘time of the day × chronotype’: F_1,16_ = .1, *p* = .76; ‘block × chronotype’: F_2,32_ = 1.22, *p* = .31; ‘time of the day × block’: F_2,32_ = .05, *p* = .95; ‘time of the day × block × chronotype: F_2,32_ = 1.13, *p* = .34). Hence, the effects of the time of the day on the mean fixation duration were independent from the chronotype of the participants *per se*.

**Figure 3:**
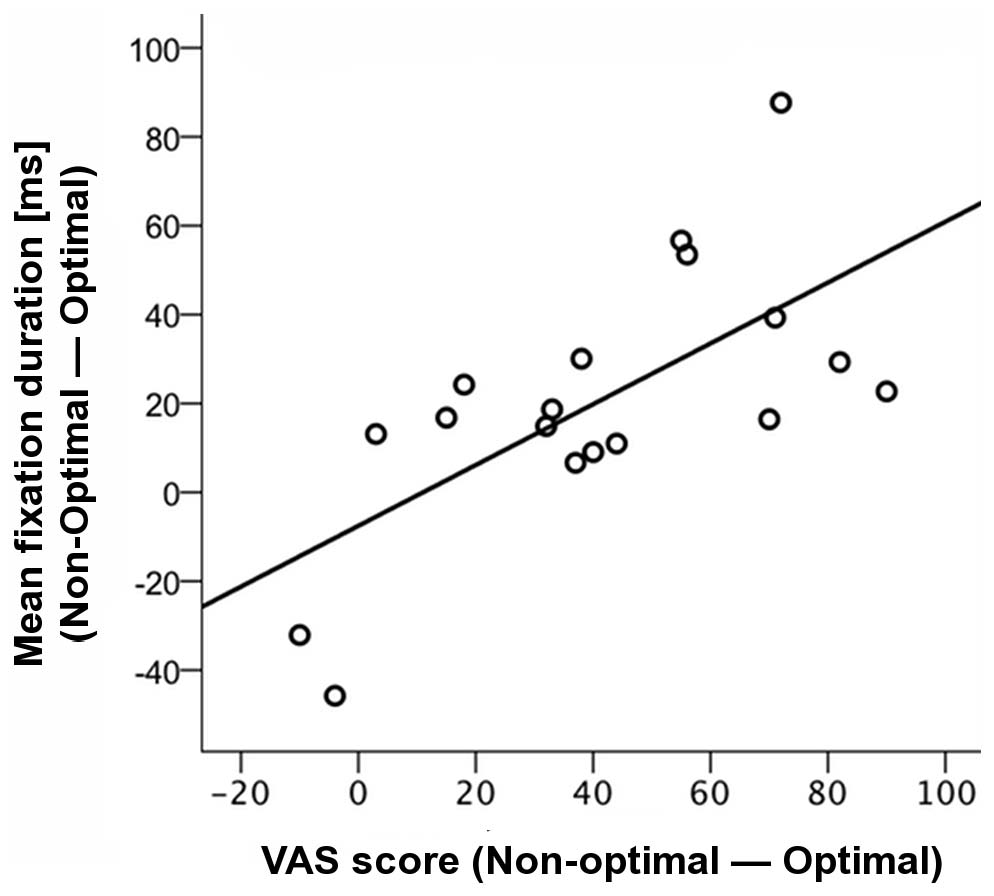
Correlation between visual analogue scale (VAS) fatigue scores and mean fixation durations. Correlation between the difference of the VAS scores and the difference of the mean fixation durations between non-optimal and optimal times of the day, with regression line.

Moreover, there was a significant correlation between the difference of the VAS scores and the difference of the mean fixation durations between non-optimal and optimal times of the day (*r* = .673, *p* = .002). The more fatigued the participants subjectively reported to be at their non-optimal time of the day, the greater was the increase in their mean fixation duration during the free visual exploration (see Fig. 3).

**Figure 4:**
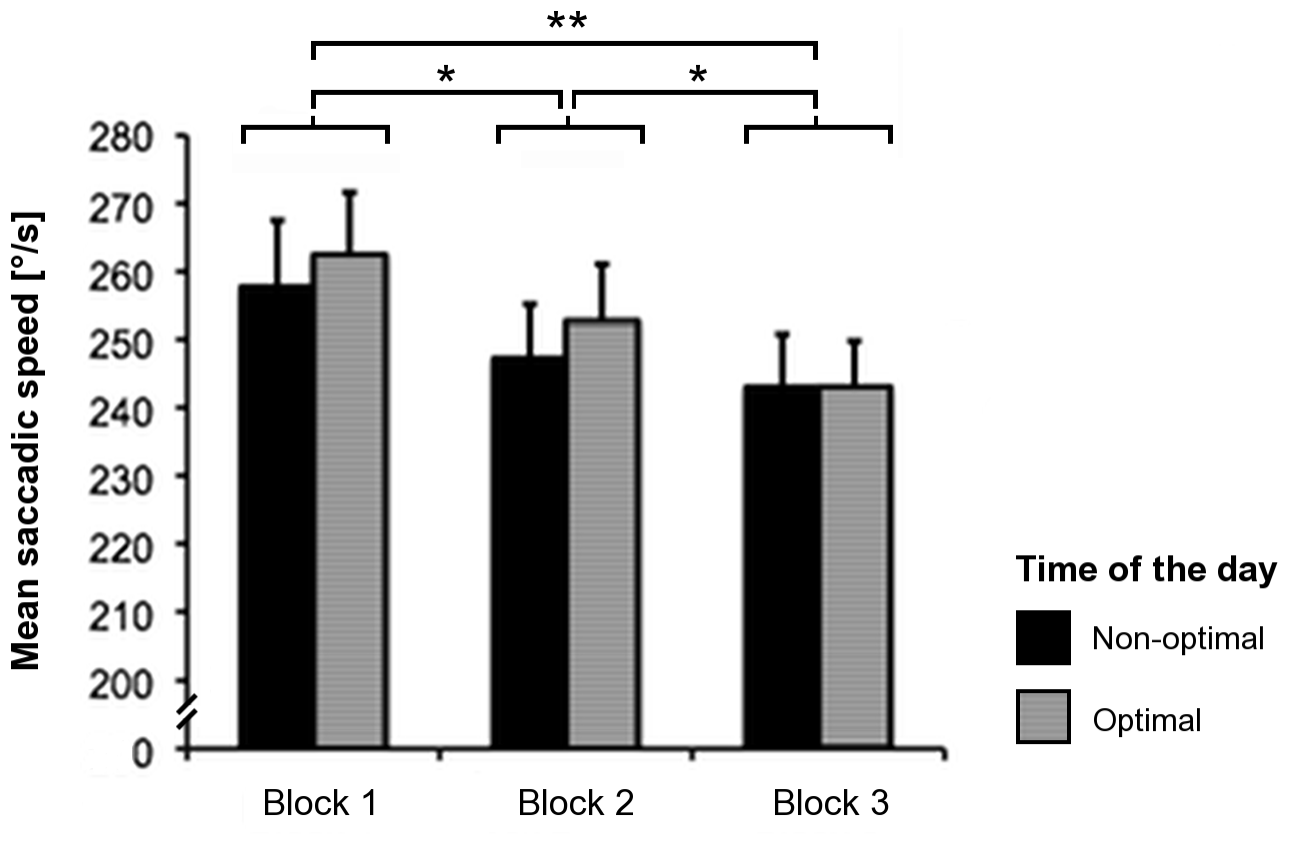
Mean saccadic speed. Mean saccadic speed in the three blocks of the free visual exploration task, as measured at optimal and non-optimal times of the day for the participants. Error bars represent the standard error of the mean (SEM). Asterisks depict significant post-hoc tests (*p<.05; **p<.01).

Concerning the mean saccadic speed during the free visual exploration, the repeated-measures ANOVA revealed a significant main effect of the factor ‘block’ (F_2,34_ = 8.71, *p* = .001), but not of the factor ‘time of the day’ (F_1,17_ = .13, *p* = .73) or of the interaction ‘time of the day × block’ (F_2,34_ = .3, *p* = .74). The mean saccadic speed during the free visual exploration significantly and progressively decreased along the different blocks of the task (see Fig. 4), independent from the optimal or non-optimal time of the day.

Rerunning the repeated-measures ANOVA with the additional factor ‘chronotype’ yielded the same results, i.e., the only significant effect was found for the factor ‘block’ (F_2,32_ = 8.66, *p* = .001), whereas the effects of the other factors or interactions were not significant (‘time of the day’: F_1,16_ = .14, *p* = .71; ‘chronotype’: F_1,16_ = .82, *p* = .38; ‘time of the day × chronotype’: F_1,16_ = 3.39, *p* = .09; ‘block × chronotype’: F_2,32_ = .83, *p* = .45; ‘time of the day × block’: F_2,32_ = .29, *p* = .75; ‘time of the day × block × chronotype: F_2,32_ = .13, *p* = .88). Hence, the progressive decrease of the mean saccadic speed during the task was independent from the chronotype of the participants *per se*.

Finally, there was no significant effect of the factor ‘time of the day’ (F_1,17_ = 3.3, *p* = .09), of the factor ‘block’ (F_2,34_ = 1.27, *p* = .29), or of the interaction ‘time of the day × block’ (F_2,34_ = .43, *p* = .65) on the mean saccadic amplitude. There is a consistent relationship between the saccadic amplitude and the saccadic speed: the larger the saccade, the greater its speed [18]. Hence, the absence of significant effects on the mean saccadic amplitude allows to exclude that the progressive decrease of the mean saccadic speed during the free visual exploration task was due to a decrease of the mean saccadic amplitude.

## Discussion

The present study shows that the non-optimal time of the day (associated with a subjectively more pronounced fatigue) triggers a significant and stable increase in the mean fixation duration during free visual exploration, irrespective of the chronotype (i.e., morning or evening type). Conversely, the mean saccadic speed significantly and progressively decreases throughout the duration of the task, but is not influenced by the optimal or non-optimal time of the day for both chronotypes.

The significant increase in mean fixation duration due to the lack of synchronicity between chronotype and time of the day is a new finding and, to the best of our knowledge, is reported here for the first time. Interestingly, the fatigue due to the lack of synchronicity between chronotype and time of the day as self-reported (by means of the VAS) correlated with the fatigue as objectively measured by a physiological parameter (by means of the mean duration of visual fixations). This is noteworthy, because correlations between self-reports and physiological measures have rarely been reported [28]. A number of studies have established that synchronicity effects trigger a clear advantage for individuals when tested at optimal as opposed to non-optimal times of the day, in particular for those tasks that involve careful cognitive processing [10], [29]. It is interesting that these synchronicity effects can also be found by means of a free visual exploration paradigm, which has high ecological (i.e., face) validity [30] and does not impose any particular constraint or pressure on the cognitive processing of the participants. On the other hand, the mean fixation duration was not influenced by the increasing time spent on task. This is in line with previous reports [22], [24], [25] and underlines the specificity of changes in the mean fixation duration as reflecting lack of synchronicity between chronotype and time of the day.

As shown in the results, there was also a gradual decrease in the mean saccadic speed with increasing time on task. This finding is in line with a number of previous studies [20], [22]. Moreover, the mean saccadic speed was not influenced by the optimal or non-optimal time of the day, underlining the specificity of changes in this parameter as reflecting the increasing time spent on the free visual exploration task.

Taken together, the results concerning mean fixation duration and mean saccadic speed outline a double dissociation in which these two oculomotor parameters are discriminative for the fatigue due to different sources. Mean fixation duration varies according to synchronicity effects, but not to the time spent on task. Inversely, mean saccadic speed varies according to the time spent on task, but not to synchronicity effects. This suggests that the fatigue experienced by the participants due to the lack of synchronicity between chronotype and time of the day is probably not based on the same mechanisms as the fatigue observed after continuous wakefulness or sleep deprivation [13], [14], [15], [16], [17]. One may interpret this dissociation as reflecting two different dynamics: a) the *process* of *getting* fatigued, interpreted as a dynamic process, developing with increasing activity or time spent on task (as reflected in a progressive decrease of mean saccadic speed due to increasing time spent on task); b) the *state* of *being* fatigued, interpreted as more stable state, independent from activity or time spent on task (as reflected in a stable increase of mean fixation durations due to the lack of synchronicity between chronotype and time of the day).

This dissociation between the effects of different fatigue sources on different oculomotor parameters could be speculatively attributed to partially distinct neuroanatomic and/or neurophysiologic substrates. Some studies have attributed the decrease in saccadic speed to energy regulation processes during the task performance [20]. As postulated by Di Stasi et al. [31], the decrease in saccadic speed might be due to a decreased excitation on the omnipause neurons (OPN). The OPN fire at constant rate during fixation, being thereby critical for the encoding of saccadic speed, and pause during saccade execution (for a review, see Girard and Berthoz [32]). A reduced or lacking pre-saccadic OPN activity is known to cause a reduction of saccadic speed (for an overview, see the chapter “Disorders of Saccadic Velocity” in [18]). The reduction of the excitatory input coming from a putative population of activity-dependent neurons on the OPN would reduce the firing rate of the latter, thus triggering a decrease in saccadic speed [31]. This might explain the effect of fatigue due to time spent on task on saccadic speed but not on fixation duration. On the other hand, a number of eye movement studies have demonstrated that the maintenance of visual fixation involves the anterior cingulate cortex (ACC) and the dorsolateral prefrontal cortex (dlPFC) [33], [34]. In addition, fixation neurons present in the frontal eye field (FEF) and the subthalamic nucleus (STN) have been shown to be tonically active during a visual fixation task [35], [36]. Fixation duration might thus be predominantly controlled by prefrontal areas and in particular, as mentioned previously, by ACC and dlPFC. Recent evidence coming from functional magnetic resonance imaging (fMRI) has shown that the activity of the dlPFC, in particular of the FEF, is modulated by the time of the day at which cognitive tasks are performed [37]. This might explain the effect of fatigue due to a lack of synchronicity between time of the day and chronotype on fixation durations but not on saccadic speed.

Finally, one might consider some limitations and possible future directions for the present study. On the one hand, the results should be replicated in a larger cohort, possibly including a broader chronotypical range (i.e., not only definitely morning or evening types). On the other hand, it would be interesting to assess the influence of activity and sleep on fatigue and oculomotor parameters, for instance by using actigraphy. Furthermore, such experiments could be conducted under more complex daily environment conditions, including, for instance, motion, sound, and behavioural goals (such as looking for specific objects in the environment).

In conclusion, the present study suggests that different oculomotor parameters, such as fixation duration and saccadic speed, may be used to discriminate fatigue from different sources, such as lack of synchronicity between time of the day and chronotype, and the process of getting fatigued.
